# Evaluation of the URGOnight Tele-neurofeedback Device: An Open-label Feasibility Study with Follow-up

**DOI:** 10.1007/s10484-021-09525-z

**Published:** 2021-09-28

**Authors:** Noralie Krepel, Tommy Egtberts, Emma Touré-Cuq, Pierre Bouny, Martijn Arns

**Affiliations:** 1 Department of Cognitive Neuroscience, Faculty of Psychology and Neuroscience, Maastricht University, Maastricht, The Netherlands; 2 Research Institute Brainclinics, Brainclinics Foundation, Nijmegen, The Netherlands; 3 NeuroCare Group Netherlands, Nijmegen, The Netherlands; 4 UrgoTech, 15 avenue d’Iéna, 75116 Paris, France

**Keywords:** Sleep problems, SMR neurofeedback (URGOnight), Feasibility trial

## Abstract

SMR neurofeedback shows potential as a therapeutic tool for reducing sleep problems. It is hypothesized that SMR neurofeedback trains the reticulo-thalamocortical-cortical circuit involved in sleep-spindle generation. As such, strengthening this circuit is hypothesized to reduce sleep problems. The current study aims to investigate the effectiveness of a home-based device that uses SMR neurofeedback to help reduce sleep problems. Thirty-seven participants reporting sleep problems received the SMR neurofeedback-based program for 40 (*n* = 21) or 60 (*n* = 16) sessions. The Pittsburgh Sleep Quality Index (PSQI) and Holland Sleep Disorders Questionnaire (HSDQ) were assessed at baseline, session 20, outtake, and follow-up (FU). Actigraphy measurements were taken at baseline, session 20, and outtake. Significant improvements were observed in PSQI Total (*d* = 0.78), PSQI Sleep Duration (*d* = 0.52), HSDQ Total (*d* = 0.80), and HSDQ Insomnia (*d* = 0.79). Sleep duration (based on PSQI) increased from 5.3 h at baseline to 5.8 after treatment and 6.0 h. at FU. No effects of number of sessions were found. Participants qualified as successful SMR-learners demonstrated a significantly larger gain in sleep duration (*d* = 0.86 pre-post; average gain = 1.0 h.) compared to non-learners. The home-based SMR tele-neurofeedback device shows the potential to effectively reduce sleep problems, with SMR-learners demonstrating significantly better improvement. Although randomized controlled trials (RCTs) are needed to further elucidate the specific effect of this device on sleep problems, this is the first home-based SMR neurofeedback device using dry electrodes demonstrating effectiveness and feasibility.

## Introduction

Neurofeedback is a therapeutic technique that seeks to modulate and retrain brain function to address neurological and/or psychological symptoms. A widely studied neurofeedback protocol, sensori-motor rhythm (SMR) neurofeedback (termed one of the ‘standard neurofeedback protocols’ (Arns et al., 2014a, 2014b)), aims to train SMR activity. SMR activity is an EEG rhythm in the low beta range (12–15 Hz) derived from the EEG, typically located over the sensori-motor strip.

SMR neurofeedback is hypothesized to train the circuit associated with the generation of sleep spindles. Sleep spindles are generally considered a hallmark of stage 2 sleep and sleep deprivation may alter sleep spindle and sigma (12–15 Hz) activity (De Gennaro & Ferrara, 2003). Sleep spindles are generated in a reticulo-thalamocortical-cortical loop. Originating from the thalamic reticular nucleus, GABAergic neurons produce spike-burst activity as a consequence of inhibitory postsynaptic potentials (IPSPs) in the thalamocortical pathway. The rebound of the IPSPs generates excitatory postsynaptic potentials in the cortical cells, which is observed as a sleep spindle (De Gennaro & Ferrara, 2003; Sinha, 2011). It was proposed that training the reticulo-thalamocortical-cortical network using neurofeedback results in long-term potentiation (LTP) which increases the synaptic strength within this network and the likelihood of its future activation (Arns & Kenemans, 2014; Sterman & Egner, 2006), suggesting that learning to control SMR activity (thus not only upregulation of SMR activity) leads to increased sleep spindle density and associated changes in sleep parameters. However, recently no effects of bidirectional (i.e., up- and downregulation) SMR training on sleep parameters were reported (Binsch et al., 2018), suggesting sleep-related improvements are specifically related to SMR neurofeedback up-training only.

Several studies have shown that SMR neurofeedback alters sleep parameters. Sterman et al. (1970) first demonstrated that training SMR activity in the awake cat, using an instrumental conditioning paradigm, resulted in increased spindle burst activity during sleep as well as longer epochs of uninterrupted sleep. Studies involving humans have reported that SMR neurofeedback resulted in significant sleep improvements in individuals with insomnia after an average of 25 sessions (Hauri, 1981; Hauri et al., 1982) as well as increased sleep spindle density and decreased sleep onset latency (SOL) in students (Hoedlmoser et al., 2008). As a follow-up to this latter study, Schabus et al. (2014) later investigated SMR neurofeedback in patients with primary insomnia and reported a reduced number of awakenings, a decreased SOL (trend-level), and an increase in slow-wave sleep after SMR neurofeedback. However, a double-blind placebo-controlled follow-up study demonstrated that sham stimulation reduced subjective sleep problems similarly to active stimulation (suggesting non-specific training effects) and a lack of improvements in objective measurements of sleep (Schabus et al., 2017). However, the intervention was confined to 12 neurofeedback sessions only, which is generally considered a low number of sessions. A home-based SMR tele-neurofeedback study in patients with primary insomnia, performing 20 sessions, resulted in a decreased SOL, less wake after sleep onset (WASO), and increased total sleep time (TST) measured using polysomnography (Cortoos et al., 2010). Concluding, studies have reported substantial improvements in sleep following neurofeedback training (similar to the original report by Sterman et al. (1970)), yet the number of sessions, especially in individuals reporting symptoms of primary insomnia, could be an influencing factor. Also, the intensive nature of neurofeedback–often requiring 30–40 sessions–makes this technique relatively expensive. Likewise, although initial positive effects of home-based SMR neurofeedback have been demonstrated by Cortoos et al. (2010), tele-neurofeedback still involves the participation of a therapist. Therefore, the current open-label study aims to investigate: 1) the feasibility of a home-based program with an EEG headset using dry EEG electrodes (URGOnight, UrgoTech, Paris); 2) the possible differential effect of 40 versus 60 sessions of neurofeedback; 3) acute and long-term (3–6-month FU) effectiveness of the intervention; and 4) whether the amount of SMR learning was associated with clinical improvements.

## Methods and Materials

This study is an open-label feasibility trial. Only patients that had a primary sleep problem and no primary psychiatric comorbidities that potentially explained the sleep problems were included. Patients between 18 and 70 years of age with a primary sleep problem expressed as a sleep onset problem (latency (SOL) ≥ 30 min), sleep maintenance problem (wake after sleep onset (WASO) ≥ 30 min), or sleep duration problem (sleeping ≤ 6 h. per night) were included. Sleep complaints had to occur at least three times a week, and the duration of complaints should be at least six months as quantified on the PSQI. Medication usage was allowed but had to remain stable during the treatment. Exclusion criteria were: comorbid medical or psychiatric complaints (as assessed using the MINI); recent parenthood; night shifts; students; pregnancy; excessive alcohol and caffeine usage; diagnosis of a primary sleep disorder other than primary insomnia.

Questionnaires assessed were the Holland Sleep Disorders Questionnaire (HSDQ), and the Pittsburgh Sleep Quality Index (PSQI). Actigraphy data were also collected (using the ActTrust (Condor Instruments; https://www.condorinst.com.br/en/acttrust-actigraph/)), where subjects wore the ActTrust for at least seven subsequent days, and pressed the event button when they had the intention to go to sleep and when they had the intention to go out of bed. Actigraphy data was analyses in the ActTrust software with default settings, and required corrections performed by the treating clinician based on the sleep–wake diary and patient feedback. All data were assessed at pre-treatment (T0), after 20 sessions (T1), and end of treatment (T2), and at FU after 3–6 months (T3, except actigraphy).

### URGOnight

**Fig. 1:**
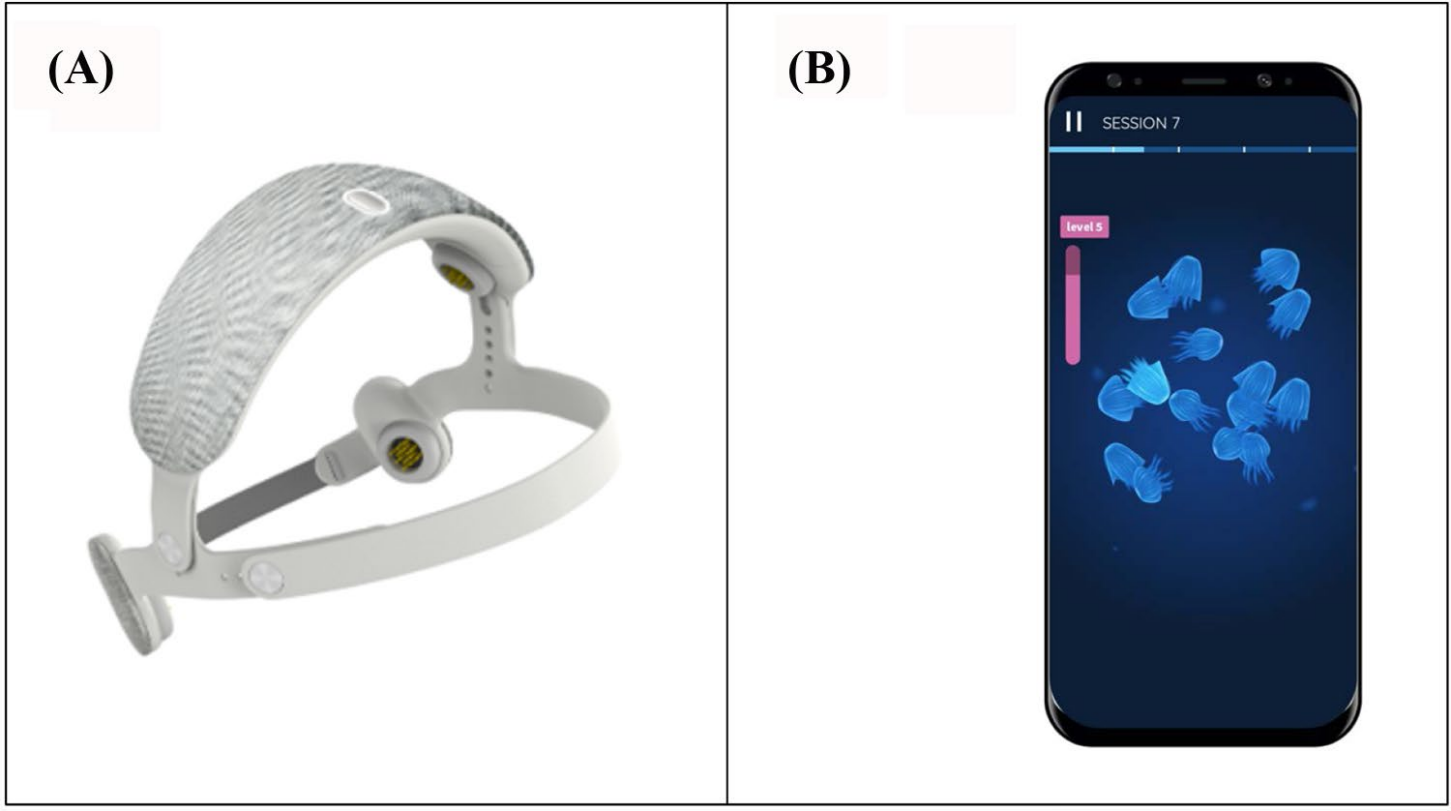
**A**-**B** URGOnight headband and URGOnight mobile app neurofeedback training session screen. **A** The headband, adjustable to head size, with two measuring dry electrodes over the sensori-motor cortex. **B** Neurofeedback training screen: the bar on the left fills in real-time when SMR power increases. The threshold is displayed by a level on top of the gage, the animated wallpaper is animated whenever SMR activity exceeds the threshold (here, the jellyfishes will illuminate and disappear as long as the participant manages to keep his or her SMR activity above threshold)

URGOnight (Fig. 1A-B) is a home-based device developed to improve sleep quality using neurofeedback and by providing sleep hygiene support. It consists of a portable EEG headband connected to a mobile application via Bluetooth technology.

The URGOnight headband includes two dry measuring electrodes (UrgoTech, Paris) over the sensorimotor cortex in positions C3 and C4 of the international 10–20 system. Reference and ground are positioned on the left and right mastoids, respectively. The EEG data measured by the headband is transferred to the mobile application in real-time to allow the user to perform neurofeedback training autonomously. In addition to the neurofeedback program, the URGOnight mobile application provides daily advice to improve sleep and sleep hygiene, an assessment of sleep quality and sleep hygiene levels using questionnaires, and a sleep diary.

The mobile application provides sleep self-monitoring with two questionnaires developed by UrgoTech: 1) a sleep quality questionnaire filled out at the start of the program, then every 10 sessions and 2) a sleep hygiene monitoring questionnaire completed at the start of the program, then at the request of the user. It also contains a simplified sleep diary that allows the user to enter their night's sleep as well as the quality of wakefulness felt during the day every day and provides the development thereof on a weekly display. Sleep advice provided by sleep experts and based on Cognitive Behavioral Therapy for Insomnia (CBTI) can also be viewed in the app, such as:

- *“Prefer a light diet for the evening: this will facilitate your digestion and your sleep (not too much, not too sweet but in good quantity). Pay attention to alcohol consumption which prevents your sleep from being restful”*
- *“Avoid practicing physical activity in the 4 h before bedtime "*

Other complementary information on neurofeedback training is also provided.

### Neurofeedback Treatment

Recording was set to a sampling frequency of 100 Hz. At home, participants trained four times per week, where every fifth session was done in the clinic supervised by a trained neurofeedback specialist. All instructions during sessions were provided by the mobile application and additional questions were answered by the therapists during weekly visits at the clinic. Neurofeedback sessions lasted approximately 20 min, including one minute of baseline measurement. This consisted of a 30-s period where participants were instructed to relax and keep their eyes closed, followed by a 30-s period with their eyes open. Then, five 3-min neurofeedback runs were performed. Participants were free to take a one-minute break between runs or to go straight to the next run.

During neurofeedback runs, participants were presented with a bar corresponding to their real-time SMR power (12–15 Hz) associated with a threshold level (1, 2, 3, 4, or 5) and an animated wallpaper. An additional character (portrayed as a robot) appeared on the screen when the EMG band power exceeded 70 μV on one of the sensors. If this was the case, the participant was asked to relax. No rewards were given when the EMG band power value exceeded the threshold.

The sound and visual environment of the training could be customized by the participants before training; however, feedback screens were designed to reflect ‘discrete’ feedback aimed at a reinforcement rate of 25–30% in line with earlier recommendations (Sherlin et al., 2011). They received audio and visual rewards each time their SMR band power value exceeded the threshold for 400 ms. When participants managed to keep their SMR power above the threshold for two seconds, the threshold was increased, and they received a visual cue to indicate that the level had increased. When SMR band power was below the threshold for seven seconds, the level was decreased to the preceding one and they received a visual cue to indicate that it had been lowered.

Participants were not instructed with a special strategy to perform the requested EEG-modulation but were told to be alert to the training and physically relaxed (Kober et al., 2013; Reichert et al., 2015). Participants were asked for self-developed strategies, some examples of which are provided below:

*“I empty my head and try to think of nothing”*

*“I fix a point in the distance”*

*“I imagine myself swimming”*

*“I count my breath and focus on my feelings”*.

To investigate the effect of a maximum number of required sessions and potential ceiling effects, half of the participants completed 40 sessions and the other half 60 sessions.

### Learner Effects

As explained in the introduction, we expect that the effects of SMR neurofeedback is about training the reticulo-thalamocortical-cortical network resulting in neuroplastic changes increasing the synaptic strength within this network and the likelihood of its future activation (Arns & Kenemans, 2014; Sterman & Egner, 2006), only visible as increased sleep spindle density during sleep (Hoedlmoser et al., 2008; Sterman et al., 1970). Therefore, we did not expect across session increases in SMR band power, but rather *within-session learning* to occur, in line with Reichert et al. (2015). The following methodology is adapted from Reichert et al. (2015) and is focused on within-session learning hypothesized to reflect the individual’s ability to successfully activate and deactivate the SMR network.

EEG power data in the alpha (8 – 12 Hz), SMR (12 – 15 Hz), beta (15 – 20 Hz), and EMG (20 – 40 Hz) bands were recorded during sessions in positions C3 and C4, extracted from local recordings in the participant’s mobile phone, and transferred to UrgoTech. The learning analysis was carried out blinded from clinical outcomes and by off-site researchers at UrgoTech and only shared for final analysis with Brainclinics upon finalization of data-analyses. Data were preprocessed and analyzed using Python (version 3.8).

The ability to perform the requested task (up-regulating SMR power during training sessions) was assessed by plotting the regression slope of normalized relative SMR power averaged across all sessions performed and for each run of training for every participant.

- Relative SMR power values were normalized with the following procedure:

o Relative SMR power in C3 = SMR / (alpha + beta + SMR + EMG)
o Relative SMR power in C4 = SMR / (alpha + beta + SMR + EMG)
o Average over electrodes: (ratio C3 + ratio C4)/2
o Z-score of average relative signal

If the participant succeeded in the task, an increase in SMR power between the first run and the fifth run (Reichert et al., 2015; Witte et al., 2013; Zoefel et al., 2011) was expected. Participants with a positive slope of regression were classified as learners, and participants with a negative slope of regression were classified as non-learners.

### Statistics

As primary outcome measure PSQI Total, PSQI Sleep Duration, HSDQ Total, and HSDQ Insomnia were defined. Secondary outcomes included the remaining subscales of the HSDQ (including parasomnia, Circadian Rhythm Sleep Disorder (CRSD), hypersomnia, Restless Legs Syndrome/Periodic Limb Movement Disorder (RLS/PLMD), and Sleep Breathing Disorder (SBD)).

To investigate if the number of sessions had an impact on the primary outcomes, repeated measures ANOVAs were performed with factor Time (pre-treatment, session 20, and post-treatment) as within-subject factor and Sample (40 vs. 60 sessions) as a between-subject factor. In case no interactions involving Sample arise, both samples will be combined, and repeated measures ANOVAs will be performed with factor Time (pre-treatment vs. post-treatment and pre-treatment vs. FU).

For Time effects a *p* < 0.01 was used to also accommodate corrections for multiple testing and a conventional *p* < 0.05 for interaction effects and other main effects. Cohen’s *d* effect sizes are calculated based on pre- vs. post-treatment effects and pre- vs. FU-treatment effects. Further sensitivity analyses carried out comprised analyses of actigraphy data and a learner analyses where learner status was added as a between-subject factor.

## Results

A total of 37 participants were included (9 male; average 48.2 yrs.), 21 of which received 40 sessions and 16 received 60 sessions of SMR neurofeedback. There were no baseline differences between males and females and age in the 40 vs. 60 session group. Of 37 participants, 10 were unmedicated, 13 used sleep medication (mostly benzodiazepines) and three used other psychoactive drugs. Clinical variables and changes over the course of the intervention are represented in Table 1. Numbers represent mean and standard deviation.

**Table 1:** Changes on various sleep scales and actigraphy from pre- to post-intervention and at FU (average FU = 5.3 months)

	Pre-treatment	Post-treatment	Follow-up
PSQI			
Global score	13.8 (3.3)	10.3 (3.9)	10.6 (4.2)
Subjective sleep duration (hrs.)	5.3 (0.9)	5.8 (1.1)	6.0 (1.1)
Sleep onset latency (m.)	43.7 (27.5)	44 (49.0)	54.1 (56.7)
HSDQ			
Global score	2.3 (0.4)	2.0 (0.4)	2.0 (0.6)
Insomnia	3.9 (0.8)	3.2 (0.9)	3.2 (1.1)
Actigraphy			
Objective sleep duration (hh:mm)	06:51	06:57	
Sleep onset latency (m)	12.6 (8.0)	11.8 (9.1)	
Sleep efficiency (%)	83.9 (9.1)	85.1 (8.9)	
Wakefulness after sleep onset (m)	58.6 (42.0)	56.4 (41.2)	

### Primary Outcome and Number of Sessions

**Fig. 2:**
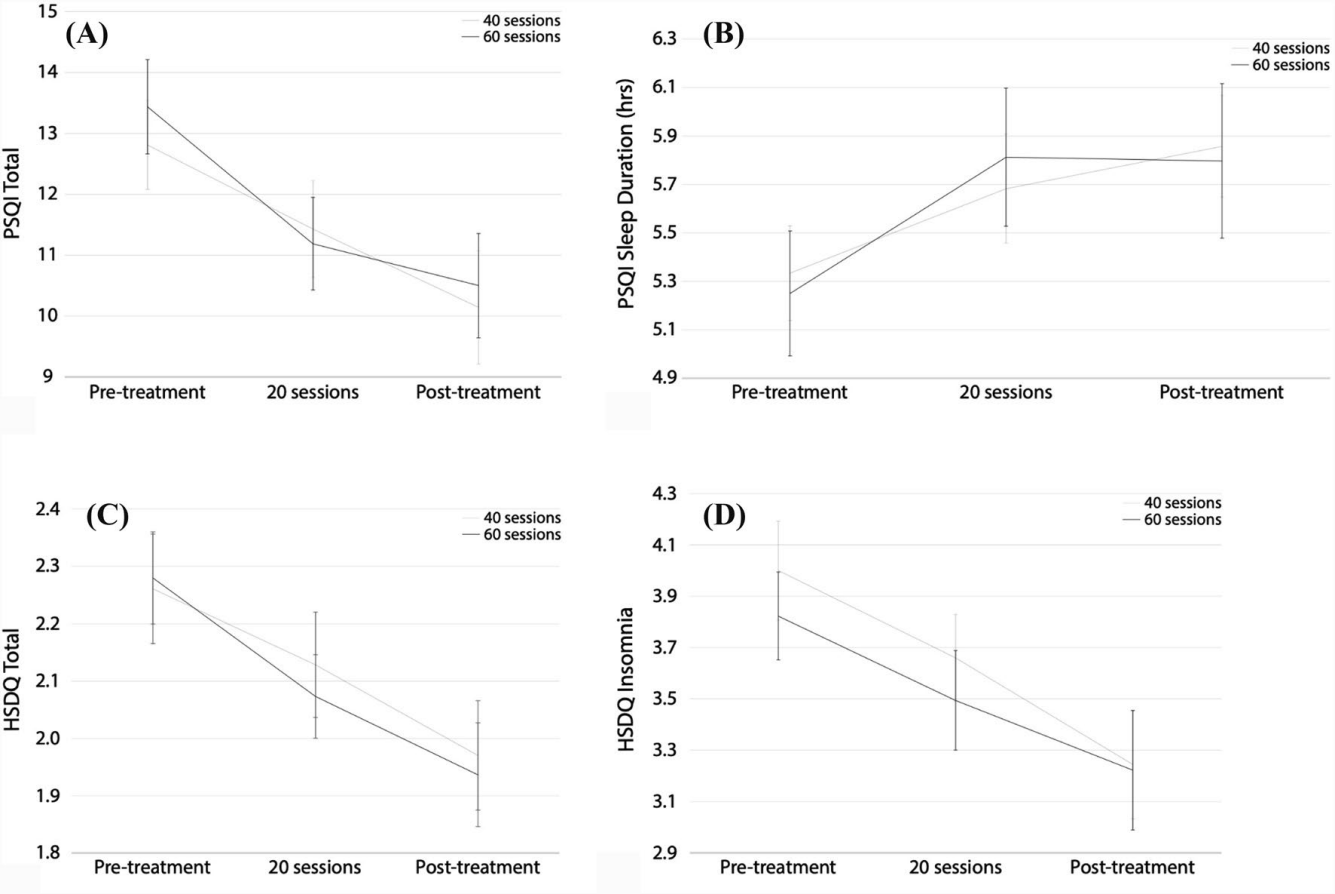
**A**–**D**: the effects on PSQI Total (**A**), PSQI Sleep Duration (**B**), HSDQ Total (**C**), and HSDQ Insomnia (**D**) over the course of treatment. Repeated measures ANOVAs using Sample (40 vs 60 sessions) as a between-subject factor and Time (pre-treatment, 20 sessions, and post-treatment) as a within-subject factor showed a significant effect of Time for PSQI Total (A: *F*(2,34) = 19.81, *p* < 0.001; *d* = 0.78), PSQI Sleep Duration (B: *F*(1,36) = 18.27, *p* < 0.001; *d* = 0.52), HSDQ Total (C: *F*(2,34) = 21.77, *p* < 0.001; *d* = 0.80), and HSDQ Insomnia (D: *F*(2,34) = 13.19, *p* < 0.001, *d* = 0.79). No interactions with Sample (*p* > 0.285) or main effects of Sample (*p* > 0.628) were observed

A repeated measures ANOVA with Time (pre-treatment, 20 sessions and post-treatment) and Sample (40 vs. 60 sessions) as a between-subject factor, yielded an effect of Time for PSQI Total (*F*(2,34) = 19.81, *p* < 0.001; *d* = 0.78 pre-post; Fig. 2A), PSQI Sleep Duration (*F*(2,34) = 10.04, *p* < 0.001; *d* = 0.52 pre-post; sleep duration increased from 5.3 h. to 5.8 h; Fig. 2B), HSDQ Total (*F*(2,34) = 21.77, *p* < 0.001; *d* = 0.80 pre-post; Fig. 2C) and HSDQ Insomnia (*F*(2,34) = 13.19, *p* < 0.001; *d* = 0.79 pre-post; Fig. 2D). No interactions with Sample (*p* > 0.285) or main effects of Sample (*p* > 0.628) were observed, suggesting no added effect of increasing the number of sessions to 60 (Fig. 2A-D). Therefore, for further analyses the 40 and 60 session groups are combined.

#### PSQI

At pre-treatment, all participants had a global PSQI score of larger than 5 (classified as ‘poor sleepers’) with an average of 13.1. This decreased to 10.3 post-treatment (*d* = 0.78) and 5/36 (13.9%) had a score of 5 or lower (classified as a. ‘healthy sleeper’).

The average gain in PSQI sleep duration was 30 min. Twenty-four participants (64.9%) improved their sleep duration with an average of 57 min, and 13 participants (35.1%) improved their subjective sleep with 1 h. or more.

#### HSDQ

At pre-treatment, 28/37 participants exceeded the HSDQ Total threshold (> 2.02) and 27/37 the Insomnia threshold (> 3.68). By the end of the intervention, only 17/37 participants exceeded the HSDQ Total threshold and 15/37 exceeded the HSDQ Insomnia threshold. This suggests a reduction to below the threshold in HSDQ Total and HSDQ Insomnia for 11/37 (29.7%) and 12/37 (32.4%) of participants, respectively. A repeated measures ANOVA with Time (pre- and post-treatment) yielded an effect of Time for HSDQ CRSD (*F*(1,36) = 20.33, *p* < 0.001; *d* = 0.65 pre-post) and HSDQ RLS/PLMD (*F*(1,36) = 10.69, *p* = 0.002; *d* = 0.37 pre-post) demonstrating decreased scores after treatment. No effects were found for subscales parasomnia, hypersomnia, and SDB (*p* > 0.220).

### Learner Effects

**Fig. 3:**
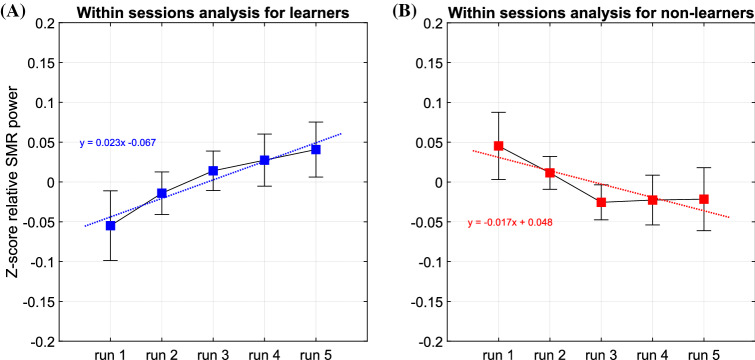
Average learning analysis regression slopes (relative SMR power z-scores within sessions, average over all sessions, error bars =  ± SD) for (**A**) learner subjects ($N=11$) who exhibit a positive slope (**B**) and non-learner subjects ($N=21$) who exhibit a negative one

Eleven participants were classified as Learners and 21 as Non-Learners, see Fig. 3 for visualization of within session learning for both groups. For the remaining five participants, no learner classification could be made due to missing session data or data recovery issues.

A repeated measures ANOVA with Time (pre- and post-treatment) as a within-subject and Learner as between-subject factor yielded an effect of Time for PSQI Sleep Duration (*F*(1,30) = 20.30, *p* < 0.001) and a Time X Learner interaction (*F*(1,30) = 5.00, *p* = 0.033). Repeating the analysis separately for non-learners yielded a non-significant effect of Time (*p* = 0.057; 5.4 to 5.75 h.) and for Learners a significant effect of Time (*F*(1,10) = 14.90, *p* = 0.003; *d* = 0.86) where sleep duration increased from 5.0 h. to 6.0 h.

The same analysis for PSQI Total, HSDQ Total and HSDQ Insomnia yielded no significant Time X Learner interaction (*p* = 0.288; *p* = 0.332; *p* = 0.396 respectively), suggesting those effects were unrelated to degree of learning.

### Follow-up Effects

For FU, data from 31 (PSQI) and 32 participants (HSDQ) were available for 3–6-month FU (average FU time: 5.3 months). A repeated measures ANOVA with Time (pre-treatment and FU) yielded an effect of Time for PSQI Total (*F*(1,30) = 30.40, *p* < 0.001; *d* = 0.83 pre-fu), PSQI Sleep Duration (*F*(1,29) = 10.56, *p* = 0.003; *d* = 0.57 pre-FU), HSDQ Total (*F*(1,31) = 56.452, *p* <  = 0.016; *d* = 0.46 pre-FU) and HSDQ Insomnia (*F*(1,31) = 15.47, *p* < 0.001; *d* = 0.81 pre-FU).

### Actigraphy

For two participants actigraphy data was unusable, thus the following data is based on 35 participants. Before treatment patients slept on average 6:51 h. which increased on the group level with six minutes to 6:57 min (*d* = 0.1).

No main effects of Time were found for Total Sleep Time (*p* = 0.632), SOL (*p* = 0.495), Sleep-Efficiency (*p* = 0.156) and WASO (*p* = 0.492).

## Discussion

The current study demonstrated that the URGOnight neurofeedback program effectively reduced sleep problems. Pre-treatment, all participants were classified as poor sleepers (based on self-reported PSQI scores) whereas 14% were classified as a ‘healthy sleeper’ post-treatment. Similar effects were observed for the HSDQ where > 78% exceeded HSDQ Total and HSDQ Insomnia thresholds pre-intervention, whereas at end of treatment 30–32% of people reduced their HSDQ Total and HSDQ Insomnia scores to below the threshold, suggesting that reductions in sleep problems were observed in approximately one-third of participants. Furthermore, sleep duration significantly increased (measured by PSQI) with a half-hour gain in sleep duration on full-group level. After an average of 5.3 months, FU results indicated maintenance of training effects without further training (Table 1) where subjective sleep duration increased further numerically (from 5.8 h. post-treatment to 6.0 h at FU). Sensitivity analyses further demonstrated that the gains in sleep duration seemed specifically confined to the learner group (with an average of increased sleep duration of 1 h), whereas the non-learner group showed a non-significant improvement of 15 min. These learner effects further suggest that the greater gains in sleep duration may be attributable specifically to the SMR neurofeedback. Finally, actigraphy data numerically supported the improvements although these effects were not significant.

SMR neurofeedback has previously been associated with decreased SOL and longer sleep duration (Arns et al., 2014a, 2014b; Cortoos et al., 2010; Hoedlmoser et al., 2008; Schabus et al., 2014), partly in line with the results reported here. One study failed to find such effects (Schabus et al., 2017), possibly due to the low number of 12 sessions. In the current study, increasing the number of sessions from 40 to 60 did not significantly affect acute or long-term outcomes in this study. This suggests that 40 sessions are sufficient to obtain clinical results and that ceiling effects are likely reached at session 40. This suggests that the optimal number of recommended sessions for SMR Neurofeedback to achieve improvements in sleep duration and sleep onset latency spans from 20 (Cortoos et al., 2010) to 40 sessions (this study).

SMR neurofeedback is hypothesized to act via the reticulo-thalamocortical-cortical sleep-spindle network. By strengthening these networks, SMR neurofeedback is thought to remediate sleep problems, expressed as a reduced SOL and increased sleep duration. In line with this hypothesis, the effects in the current study are primarily seen on sleep onset- or duration-related measures such as insomnia and CRSD. Interestingly, no significant effects were found for other sleep-related issues (SBD, hypersomnia, and parasomnia). Further specificity of the findings is derived from the specific effect in the learner group, where learners improved sleep duration significantly with one hour, and a non-significant improvement for non-learners. On the other hand, non-specific and placebo effects cannot be ruled out, thus future studies with placebo-control and polysomnography should further elucidate the specificity of effects and the proposed working mechanism.

In comparison to Cortoos et al. (2010), the severity of sleep problems for the group in this study was slightly higher (PSQI_Cortoos_ = 11.7; PSQI_this study_ = 13.4), albeit the average age of the current sample was also higher (average 49.1 yrs. vs 42–44 yrs. in Cortoos et al. (2010)). A further important difference is that in this study only SMR power was uptrained, with no downtraining or inhibits on theta and higher beta, as was done in prior studies including Cortoos et al. (2010). The total sleep gain reported by Cortoos et al. (2010) was 44 min for the neurofeedback group, a bit more than the 30 min sleep gain reported in this study. Also, the objective improvement as measured with actigraphy in this study was smaller (6 min). The dissociation between objective and subjective sleep measures is a known issue in sleep medicine and has been often reported in individuals with sleep problems (Bianchi et al., 2013; Rezaie et al., 2018), as well as in healthy individuals (Baker et al., 1999). To further elucidate the working mechanism, future studies should complement this by using the gold-standard polysomnography to more accurately quantify sleep and to track changes in sleep-spindle density as a mediator of treatment effect, using sufficient neurofeedback sessions.

Limitations include the lack of a control group, although this study was designed as an open-label feasibility study. The results from this study could inform future (randomized controlled) studies, including a placebo control and inform future power calculations for home-based tele-neurofeedback applications, where also more formal corrections for multiple testing can be adequately powered for. Another limitation includes the relatively small sample size in the learner analyses (*n*_Learners_ = 11). Further research should investigate the effects of the training on objective sleep parameters measured with polysomnography as well as the hypothesized physiological mechanism mediated by sleep spindles.

To summarize, this study demonstrates the feasibility of a home-based SMR tele-neurofeedback intervention, and the device tested in this study demonstrated the potential to effectively reduce sleep problems, with SMR-learners demonstrating significantly better improvement by 1 h. average sleep gain compared to non-learners.

## Data Availability

Not applicable.
